# Computational peptidology approach to the study of the chemical reactivity and bioactivity properties of Aspergillipeptide D, a cyclopentapeptide of marine origin

**DOI:** 10.1038/s41598-021-04513-z

**Published:** 2022-01-11

**Authors:** Norma Flores-Holguín, Juan Frau, Daniel Glossman-Mitnik

**Affiliations:** 1 Laboratorio Virtual NANOCOSMOS, Departamento de Medio Ambiente y Energía, Centro de Investigación en Materiales Avanzados, 31136 Chihuahua, CHIH Mexico; 2 Departament de Química, Universitat de les Illes Balears, Palma de Mallorca, 07122 Spain

**Keywords:** Computational chemistry, Density functional theory, Quantum chemistry, Chemistry, Cheminformatics, Computational chemistry, Drug discovery and development

## Abstract

Aspergillipeptide D is a cyclic pentapeptide isolated from the marine gorgonian Melitodes squamata-derived fungus Aspergillus sp. SCSIO 41501 that it has been shown to present moderate activity against herpes virus simplex type 1 (HSV-1). Thus, this paper presents the results of a computational study of this cyclopentapeptide’s chemical reactivity and bioactivity properties using a CDFT-based computational peptidology (CDFT-CP) methodology, which is derived from combining chemical reactivity descriptors derived from Conceptual Density Functional Theory (CDFT) and some Cheminformatics tools which may be used. This results in an improvement of the virtual screening procedure by a similarity search allowing the identification and validation of the known ability of the peptide to act as a possible useful drug. This was followed by an examination of the drug’s bioactivity and pharmacokinetics indices in relation to the ADMET (Absorption, Distribution, Metabolism, Excretion, and Toxicity) characteristics. The findings provide further evidence of the MN12SX density functional’s superiority in proving the Janak and Ionization Energy theorems using the proposed KID approach. This has proven to be beneficial in accurately predicting CDFT reactivity characteristics, which aid in the understanding of chemical reactivity. The Computational Pharmacokinetics study revealed the potential ability of Aspergillipeptide D as a therapeutic drug through the interaction with different target receptors. The ADMET indices confirm this assertion through the absence of toxicity and good absorption and distribution properties.

## Introduction

There is no doubt that peptides are among the most important biomolecules in nature. Peptides are nitrogen and amino acid sources that are linked to a variety of physiological processes. Because of its astonishing range of structures and useful functionalities, this class of chemicals has gotten a lot of attention^1^.

Based on their structural characteristics, amino acid composition, and sequences, several marine peptides from sources such as seaweeds, fishes, mollusks, crustaceans, crabs, and marine bacteria and fungi show a variety of biological activities such as antitumor, antimicrobial, antivirus, antioxidant, and antiinflammatory effects, as well as other pharmaceutical properties^2,3^. Fungal marine microorganisms are a valuable source of bioactive natural products. Proteins and peptides from marine fungi show minimal human toxicity and less adverse effects comparable to synthetic drugs^4^.

Cyclopeptides are polypeptide chains arranged in a circular sequence between amino acids forming amide bonds. Cyclic peptides show favorable characteristics, such as low toxicity, good binding affinity, and target selectivity, that make them attractive candidates for the development of therapeutic drugs. Cyclopeptides are more cell permeable and have better biological activity compared with their linear counterparts due to their reduced conformational flexibility^1^. Many studies have demonstrated that marine cyclopeptides have a wide range of biological effects, including anticancer, anthelmintic, insecticidal, antibiotic, antifungal, immunosuppressive, anti-inflammatory, anti-HIV, and anti-malarial properties^5–12^.

A cyclic pentapeptide named Aspergillipeptide D was isolated from the South China Sea gorgonian Melitodes squamata-derived fungus Aspergillus sp. SCSIO 41501 with moderate activity against herpes virus simplex type 1 (HSV-1)^13^. It also showed antiviral activity towards acyclovir-resistant clinical isolates of HSV-1-106 and HSV-1-153^14–16^.

As a follow up of our previous studies on the chemical reactivity properties of marine cyclopeptides^17–25^, we think that it is worth to report the physicochemical and bioactivity properties of the cyclic pentapeptide Aspergillipeptide D as well as to predict and understand its chemical reactivity properties through a methodology developed by our group as a means of further validation of the procedure, while at the same time assessing the behavior of different density functionals in fulfiling the Janak and Ionization Energy theorems the KID methodology and the Ionization Energy Theorem, which is a corollary of Janak’s theorem^26–30^.

Thus, the objective of this work is to report the results of a computational study of the bioactivity properties and chemical reactivity of this cyclopentapeptide regarding a CDFT-based computational peptidology (CDFT-CP) methodology^17–25^ caused by the combination of the chemical reactivity descriptors which emanate from Conceptual Density Functional Theory (CDFT)^31–36^ with some Cheminformatics tools^37–44^ which may be utilized to assess the associated physicochemical properties to enhance the virtual screening procedure and to detect the peptide’s ability to act as a possible useful drug, supplemented with an analysis of its bioactivity and pharmacokinetics characteristics linked to the ADMET features^45–47^.

## Methods

### Computational pharmacokinetics analysis and ADMET study

The SMILES notation of the cyclopeptide acquired by accessing ChemDoodle 11.3.0 software, was fed into the online program Chemicalize from ChemAxon (http://www.chemaxon.com), which was utilized for naming and to get a glimpse of the potential therapeutic properties of the considered cyclic pentapeptide (date of access: March 2021).

A similarity search in the chemical space of compounds with molecular structures that could compared to the one that is being studied with already known biological and pharmacological properties was achieved through the online Molinspiration software from Molinspiration Cheminformatics (https://www.molinspiration.com/) (accessed, March 2021).

SwissTargetPrediction is an online tool for predicting protein targets of small compounds, and it was used to determine the potential bioactivity of the marine cyclopentapeptide studied in this study citeDaina2019. The accompanying website allows for the prediction of a small molecule’s most likely macromolecular targets, assuming it is bioactive.

Pharmacokinetics is a procedure that involves determining the likely fate of a medicinal molecule in the body, which is critical information in the creation of a new medicine. Individual indices named Absorption, Distribution, Metabolism, Excretion, and Toxicity (ADMET) factors have typically been used to analyze the associated consequences. Chemicalize and the internet available SwissADME program were used to estimate some ADMET parameters in this study^45^. pkCSM, a software for the prediction of small-molecule pharmacokinetic properties using SMILES that can be accessed through its linked webpage, was used to gain additional information regarding the Pharmacokinetics parameters and ADMET qualities^46^.

### Density functional theory (DFT) calculations

The Kohn–Sham (KS) methodology involves the electronic density, the determination of the molecular energy, and the orbital energies of a specific system, in particular, the HOMO and LUMO frontier orbitals which are intrinsically related to the chemical reactivity of the molecules^48–51^. This methodology is convenient when thinking of quantitative qualities related with Conceptual DFT descriptors^31–36^. The definitions for the global reactivity descriptors are^31–36^: Electronegativity as $\chi \approx \frac{1}{2} (\epsilon _L + \epsilon _H)$, Global Hardness as $\eta \approx (\epsilon _L - \epsilon _H)$, Electrophilicity as $\omega \approx {(\epsilon _L + \epsilon _H)^2}/{4 (\epsilon _L - \epsilon _H)}$, Electrodonating Power as $\omega ^{-} \approx {(3 \epsilon _H + \epsilon _L)^{2}}/{16 \eta }$, Electroaccepting Power as $\omega ^{+} \approx {(\epsilon _H + 3 \epsilon _L)^{2}}/{16 \eta }$ while the Net Electrophilicity is $\Delta \omega ^{\pm } = \omega ^{+} + \omega ^{-}$, being $\epsilon _H$ and $\epsilon _L$ the frontier orbital energies related to the marine cyclopentapeptide considered in this research.

These global reactivity descriptors that arise from Conceptual DFT^31–36^, has been complemented by a Nucleophilicity Index N^52–56^ that takes into account the value of the HOMO energy obtained by means of the KS scheme using an arbitrary shift of the origin with tetracyanoethylene (TCE) as a reference.

The density functional quality may be obtained by comparing its results with results from high-level computations or from experiential values. Nevertheless, this comparison is not always computationally practicable because of the large size of the molecules or the lack of experimental results for the chemical methods being explored. Our research group has developed a methodology known as KID^21–25^, in order to evaluate a particular density functional with regard to its internal coherence. It is evident that within the Generalized Kohn–Sham (GKS) version of DFT, some relationships exist between the KID methodology and the Ionization Energy Theorem, which is a corollary of Janak’s theorem^26–30^. This is done by connecting $\epsilon _H$ to -I and $\epsilon _L$ to -A, through $J_{I}$ = $\epsilon _H$ + $E_{gs}$(N − 1) − $E_{gs}$(N), $J_{A}$ = $\epsilon _L$ + $E_{gs}$(N) − $E_{gs}$(N + 1), and $J_{HL}$ = $\sqrt{{J_{I}}^2 + {J_{A}}^2}$. Another KID descriptor $\Delta$SL related to the difference in energies between the SOMO and the LUMO of the neutral system has been devised to aid in the verification of the accuracy of the methodology^21–25^.

The conformers of the cyclic peptide were established using MarvinView 17.15 from ChemAxon (http://www.chemaxon.com), which was applied in order to undertake Molecular Mechanics calculations utilizing the complete MMFF94 force field^57–61^. This was followed by a geometry optimization and frequency calculation by means of the Density Functional Tight Binding (DFTBA) methodology^62^. This last step was required for the verification of the absence of imaginary frequencies to confirm the stability of the optimized structure as being a minimum in the energy surface. The determination of the electronic properties and the reactivity descriptors of Aspergillipeptide D addressed the MN12SX/Def2TZVP/H2O model chemistry^63–65^ because it has been previously shown that it authenticates the KID procedure and satisfies the Ionization Energy Theorem^21–25^, with the aid of the Gaussian 16 software^62^ and the SMD solvation model^66^. This model chemistry considers the MN12SX screened-exchange density functional^63^ together with the Def2TZVP basis set^64,65^ and the molecule’s charge being zero whereas the radical anion and cation were considered in the doublet spin state. The SMD solvation model was chosen because it has been shown^21–25^ that it provides atomic charges of the Hirshfeld type that are almost independent of the basis set and which are usually recommended for calculations within Conceptual Density Functional Theory.

## Results and discussion

**Figure 1:**
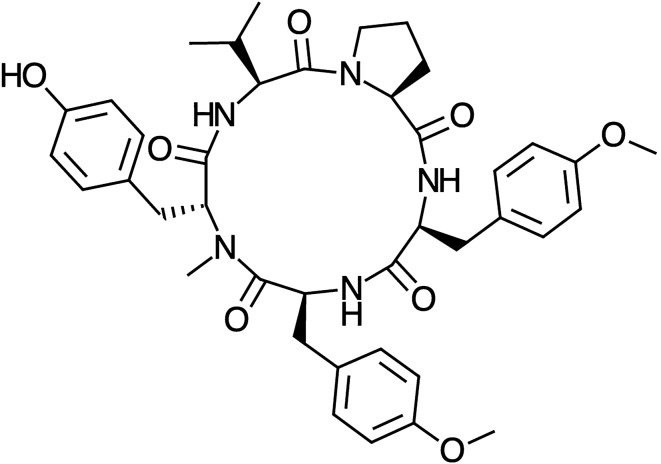
Graphical sketch of the molecular structure of the Aspergillipeptide D marine cyclopentapeptide.

ChemSpider (https://www.chemspider.com), a free chemical structure database containing information on physical, chemical, and biological properties, interactive spectra, and literature references, was used to derive the beginning molecular structure of the investigated marine cyclopentapeptide. Figure 1 shows a graphical representation of Aspergillipeptide D’s chemical structure:

### Names, identifiers and physicochemical properties

The names, identifiers and basic properties of the Aspergillipeptide D marine cyclopentapeptide are presented in Table 1.

**Table 1:** Names, identifiers and basic properties of the Aspergillipeptide D marine cyclopentapeptide.

Property	Value
Common name	Aspergillipeptide D
ChemSpider ID	61360299
Molar mass	727.859 g/mol
Exact mass	727.358113558 Da
Formula	$\hbox {C}_{{40}}$H$_{49}$N$_{5}$O$_{8}$
Composition	C (66.01%), H (6.79%), N (9.62%), O (17.58%)
IUPAC name	(3S,6S,9R,12S,17aR)-9-[(4-hydroxyphenyl)methyl]-3,6-bis[(4-methoxyphenyl)methyl]-8-methyl-12-(propan-2-yl)-hexadecahydro-1H-pyrrolo[1,2-a]1,4,7,10,13-pentaazacyclopentadecane-1,4,7,10,13-pentone
SMILES	COC1=CC=C(C[C@@H]2NC(=O)[C@H]3CCCN3C(=O)[C@@H](NC(=O) [C@@H](CC3=CC=C(O)C=C3)N(C)C(=O)[C@H(CC3=CC=C(OC)C=C3) NC2=O)C(C)C)C=C1
InChIKey	GXJNHGQMYBROHH-NGFPHSLCSA-N

This information could be useful for future QSAR investigations based on the peptide, as well as prospective Peptidomimetics derivatives created for therapeutic purposes.

### Chemoinformatics and bioactivities

**Figure 2:**
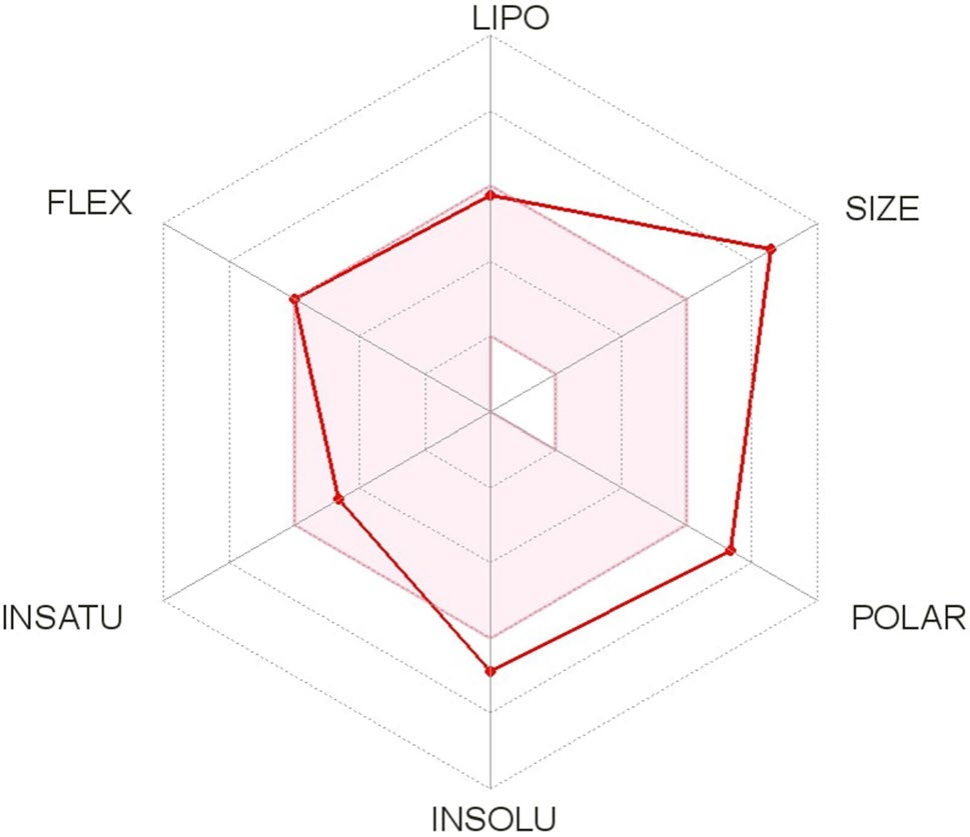
Bioavailability radar of Aspergillipeptide D.

For the Aspergillipeptide D marine cyclopentapeptide, a compact depiction of the characteristics linked to bioavailability can be displayed in a pictorial fashion through the so-called Bioavailability Radar illustrated in Fig. 2.

It is understood that the only difficulty for the Aspergillipeptide D marine cyclopentapeptide to be considered as a therapeutic drug of wide bioavailability is that considering its size and polarity whose values are somewhat larger than the ideal ones.

**Figure 3:**
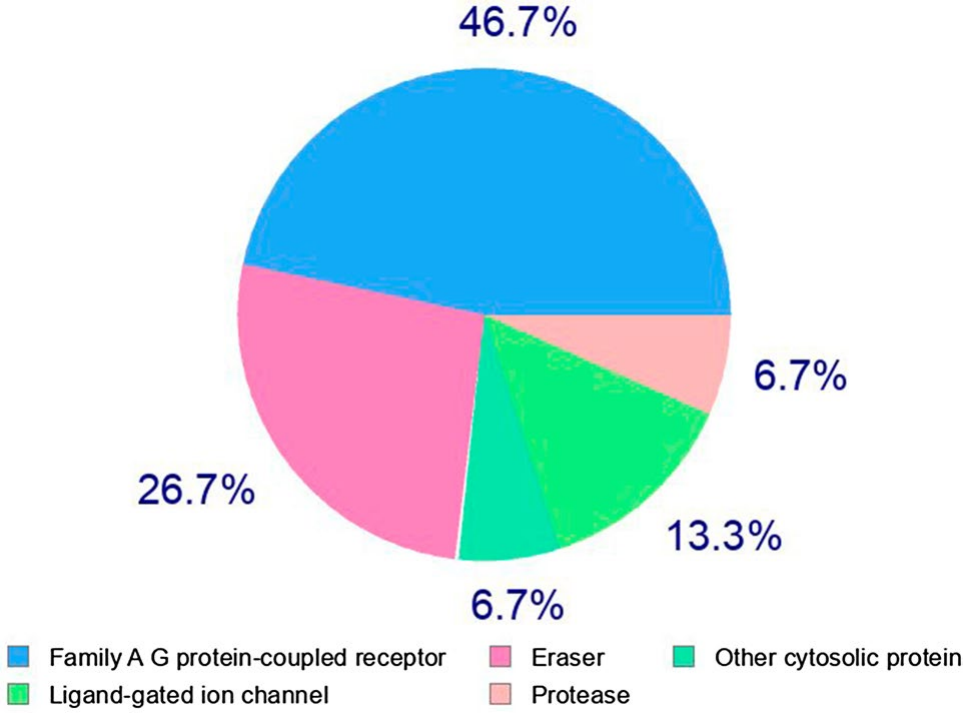
Predicted biological targets of Aspergillipeptide D.

The majority of medicinal chemicals work by attaching to target protein molecules and modifying their function. The Bioactivity Scores, which are a measure of a molecule’s capacity to act or coordinate with distinct receptors, for the Aspergillipeptide D marine cyclopentapeptide are listed in Table 2, with a graphical representation in Fig. 3 as the Biological Targets.

**Table 2:** Bioactivity scores of the Aspergillipeptide D marine cyclopentapeptide.

Property	Value
GPCR ligand	− 0.81
Ion channel modulator	− 2.00
Nuclear receptor ligand	− 1.58
Kinase inhibitor	− 1.65
Protease inhibitor	− 0.28
Enzyme inhibitor	− 1.33

The main conclusion from the results of Table 2 and Fig. 3 is that the Aspergillipeptide D marine cyclopentapeptide will exert its ability as therapeutic drug mainly behaving as a GPCR ligand and a protease inhibitor.

The pharmacokinetics of a drug is evaluated by ADMET research, which represents Absorption, Distribution, Metabolism, Excretion, and Toxicity. If absorption is unsatisfactory, the distribution and metabolism of the drug would be changed, potentially resulting in nephrotoxicity and neurotoxicity. The objective of the research is to figure out how a drug molecule behaves within an organism. As a result, ADMET analysis is one of the most important aspects of computational drug design.

The computed ADMET properties of the Aspergillipeptide D marine cyclopentapeptide are presented in Table 3.

**Table 3:** ADMET properties of the Aspergillipeptide D marine cyclopeptide.

Property	Value
**Absorption**	
Water solubility	− 3.589
Caco-2 permeability	0.610
Intestinal absorption	67.99
Skin permeability	− 2.735
P-gp substrate	Yes
P-gp I inhibitor	Yes
P-gp II inhibitor	Yes
**Distribution**	
VDss (human)	− 0.210
Fraction unbound	0.023
BBB permeability	− 0.650
CNS permeability	− 3.216
**Metabolism**	
CYP	
2D6 substrate	No
3A4 substrate	Yes
1A2 inhibitor	No
2C19 inhibitor	No
2C9 inhibitor	No
2D6 inhibitor	No
3A4 inhibitor	Yes
**Excretion**	
Total clearance	0.101
Renal OCT2 substrate	No
**Toxicity**	
AMES toxicity	No
MRTD	0.178
hERG I inhibitor	No
hERG II inhibitor	Yes
ORAT	2.737
ORCT	3.448
Hepatotoxicity	Yes
Skin sensitization	No
*T. pyriformis* toxicity	0.285

A chemical can reach a tissue if it is injected into the bloodstream. Before being taken up by target cells, a drug is usually given through mucous surfaces such as the digestive tract, i.e. intestinal absorption. Drug absorption is limited following oral delivery due to poor substance solubility, intestinal transit time, gastric emptying time, difficulty permeating the intestinal wall, and chemical instability in the stomach. Absorption is important because it affects the bioavailability of a chemical. For medications with low absorption, oral delivery, such as inhalation or intravenously, is less desirable^46,67^.

For projected values > 0.90, a substance is deemed to have a high Caco-2 permeability across the human intestinal mucosa, giving the Aspergillipeptide D marine cyclopentapeptide a value lower than the ideal. In most cases, the gut is the principal location of medication absorption from an orally delivered solution. Intestinal Absorption forecasts the percentage of a substance that will be absorbed through the human intestine, with less than 30% being considered poorly absorbed. The Aspergillipeptide D marine cyclopentapeptide should be well absorbed, according to Table 3. The model forecast whether or not a particular substance will be a P-glycoprotein substrate. For the Aspergillipeptide marine cyclopentapeptide, the prognosis is optimistic. Modulation of P-glycoprotein-mediated transport has significant pharmacokinetic implications for P-glycoprotein substrates, which might have therapeutic benefits or create contraindications. As a result, this study indicates that all of the marine cyclopentapeptides studied will inhibit P-glycoprotein I and II. Furthermore, it may be predicted whether a certain substance will be skin permeable. If a chemical has a log Kp > − 2.5, it is regarded to have low skin permeability, meaning that Aspergillipeptide D will not be useful in the development of transdermal medication administration^46^.

The total dose of a drug requires a certain volume to be uniformly distributed in blood plasma known as VDss. The drug will be more distributed in the tissue rather than in the plasma for higher VDss. From Table 3, a low value of VDss is found for Aspergillipeptide D. The efficacy of a given drug may be affected by the degree to which it binds proteins within the blood. The Fraction Unbound predicts the fraction that will be unbound in plasma resulting in the value shown in Table 3. A drug’s ability to cross into the brain is a significant descriptor because it will be able to contribute to the reduction of toxicities and side effects, and is evaluated through the Blood–Brain Permeability parameter. For a given potential therapeutic drug, a logBBB > − 0.3 value is estimated to readily cross the blood–brain barrier while molecules with logBBB > − 1 will be badly distributed to the brain. The CNS Permeability is another measurement having a value of − 3.216 forecasted for the Aspergillipeptide D which indicates that this drug cannot penetrate the Central Nervous System (CNS)^46^.

Cytochrome P450 is an important detoxification enzyme in the body, mostly present in the liver, since it oxidizes xenobiotics to enhance excretion^46^. Table 3 shows that, with the exception of CYP3A4, the Aspergillipeptide D marine cyclopentapeptide is anticipated to be non-inhibitory to all P450 cytochrome isoforms. It is also critical to be aware if a medicine is a cytochrome P450 substrate. The prediction suggests that this will not apply for CYP2D6, but rather for CYP3A4.

Drug clearance happens as a combination of renal and hepatic clearance, and is associated with bioavailability; consequently, it is important for determining dosing rates. The forecasted Total Clearance of Aspergillipeptide D is given in log(ml/min/kg). OCT2 is a renal uptake transporter which occupies an important function clearance the kidneys and in drug disposition. The cyclopentapeptide considered in this investigation has a mimimum potential to act as an OCT2 substrate, according to the results^46^.

The AMES toxicity test utilises microbes in oder to ascertain a compound’s mutagenesis potential. A positive test shows that the substance is mutagenic; therefore, it could result in cancer. The prediction is negative for the cyclopentapeptide under study. The maximum recommended tolerated dose (MRTD) is a measure of a chemical’s hazardous dosage threshold in humans. Aspergillipeptide D has a low MRTD. The main causes of acquiring long QT syndrome are the blocking of the potassium channels encoded by hERG, which leads to fatal ventricular arrhythmia. The predictions indicate that Aspergillipeptide D is unlikely to be a hERG I inhibitor but the opposite will be for hERG II. The lethal dosage value (LD50) can be assessed in terms of the ORAT (Oral Rat Acute Toxicity) and the ORCT (Oral Rat Chronic Toxicity) parameters. Drug-induced liver injury is a major safety concern for drug development and a significant cause of drug attrition. Thus, Hepatoxicity is related to the disruption of the normal liver function and the prediction for Aspergillipeptide D is positive. from another perspective, the prediction for Skin Sensitization is negative. *T. pyriformisis* is a protozoa bacteria whose toxicity is frequently applied as a toxic endpoint. A forecasted value > − 0.5 for a given compound is considered toxic^46^.

### Conceptual DFT calculations

**Figure 4:**
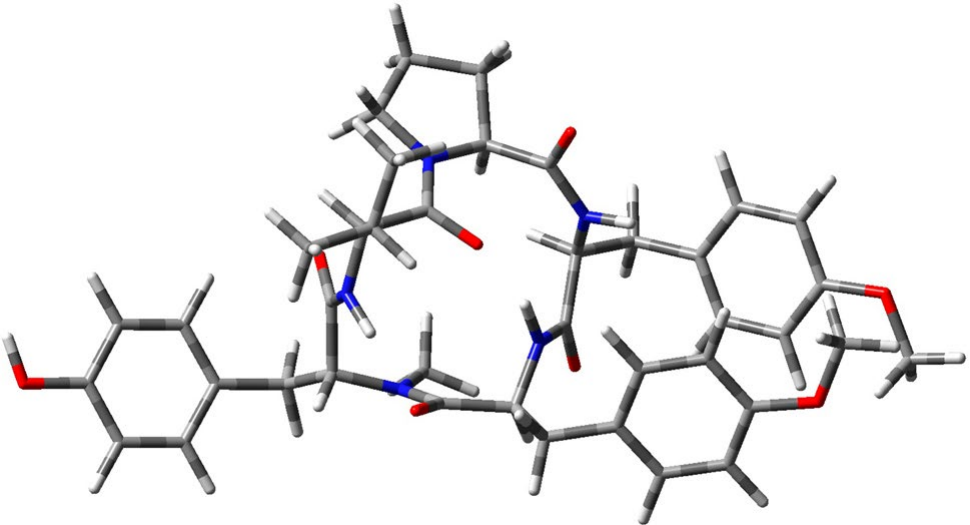
Optimized molecular structure of Aspergillipeptide D.

The optimized molecular structure of the Aspergillipeptide D marine cyclopentapeptide is computed in accordance with the process shown in the Materials and Methods section as displayed in Fig. 4.

The MN12SX density functional has been shown to have a Koopmans-compliant behavior in earlier peptides studies^17–25^ However, we believe that further validation of the marine cyclopentapeptide under investigation is necessary. The CDFT software tool was used to make this determination, and the findings are shown in Table 4. A recent study^30^ has contrasted such behavior with a group of density functionals that includes the usual B3LYP^68–70^ and PBE0^71,72^ density functionals, the local density functionals BLYP^69,70,73,74^ and PBE^75^ together with their long-range corrected variants, LC-BLYP and LC-PBE^76^, three longe-range corrected density functionals, CAM-B3LYP^77^, LC-$\omega$HPBE^78^ and $\omega$B97XD^79^, as well as three recently proposed density functionals, RSX-PBE, RSX-PBE0 and RSX-PBE0-1/3^80^. In order to attain completeness, Table 4 shows a comparison of the fulfillment of the Ionization Energy theorem between the aforementioned density functionals and the screened-exchange MN12SX density functional used in this and previous cyclopeptides research:

**Table 4:** The orbital energies (HOMO, LUMO and SOMO), the resulting HOMO-LUMO gap and the KID indices (all in eV) for the Aspergillipeptide D marine cyclopentapeptide.

Density functional	HOMO	LUMO	SOMO	H-L gap	J$_I$	J$_A$	J$_{HL}$	$\Delta$SL
B3LYP	− 6.01	− 0.62	− 1.05	5.39	0.15	0.22	0.27	0.43
PBE0	− 6.32	− 0.50	− 1.19	5.82	0.26	0.34	0.43	0.69
LC-BLYP	− 8.80	1.87	− 3.49	10.67	2.70	2.60	3.74	5.36
LC-PBE	− 9.06	1.63	− 3.78	10.69	2.71	2.63	3.78	5.41
MN12SX	− 6.09	− 1.04	− 1.13	5.05	** 0.02**	** 0.03**	** 0.03**	** 0.09**
CAM-B3LYP	− 7.43	0.72	− 2.07	8.16	1.48	1.31	1.97	2.79
LC-$\omega$HPBE	− 8.75	1.70	− 3.53	10.45	2.60	2.55	3.64	5.23
$\omega$B97XD	− 7.43	0.73	− 2.07	8.16	1.48	1.31	1.97	2.79
RSX-PBE	− 9.01	1.60	− 3.73	10.61	2.67	2.60	2.60	3.73
RSX-PBE0	− 9.04	1.64	− 3.74	10.67	2.72	2.61	3.77	5.38
RSX-PBE0-1/3	− 9.06	1.65	− 3.75	10.71	2.74	2.63	3.79	5.40

Data in bold indicates values of significance.

From Table 4, the values for the KID descriptors are all very close to zero for the MN12SX density functional meaning that it is the only one that fulfils the Janak and Ionization Energy theorems further justifying that the MN12SX/Def2TZVP/H2O is a model chemistry which is of particular relevance to this research.

During the calculation of the chemical reactivity descriptors, it is necessary to resort to the HOMO and LUMO frontier orbital energies. As shown in Table 4, the MN12SX density functional is the best one in fulfilling the Janak and Ionization Energy theorems. Indeed, these theorems only speak about the Ionization Energies. That is, the energy of the HOMO must be equal to the Ionization Energy of the molecule. Indeed, in principle, this is not strictly valid for the LUMO being equal to the electron affinity. Due to this, the HOMO-LUMO gap differs from the bandgap in a quantity that is called Derivative Discontinuity Energy (DDE). However, the electron affinity will be equal to energy of the SOMO, which is the HOMO of the radical anion. Our defined accuracy descriptor $\Delta$SL amounts for the difference in energy between the LUMO and the SOMO. As it can be observed from Table 4, for the MN12SX density functional the value of $\Delta$S (shown in bold) is very close to zero. This result has the implication that the LUMO and the SOMO energies will be almost the same and that the electron affinity of the molecule is accurately represented by the LUMO energy. Additionally, the DDE will be negligible implying that the HOMO, LUMO and HOMO-LUMO gap energies will be predicted with great accuracy allowing an excellent estimation of the chemical reactivity descriptors derived from Conceptual DFT.

The defined global reactivity descriptors’ values (including the Nucleophilicity N) for the Aspergillipeptide D marine cyclopentapeptide acquired utilizing the mentioned CDFT tool are displayed in Table 5.

**Table 5:** Global reactivity descriptors for the Aspergillipeptide D marine cyclopentapeptide (all in eV, with the exception of softness, eV$^{-1}$).

Descriptor	Value
Electronegativity ($\chi$)	3.5617
Global hardness ($\eta$)	5.0483
Electrophilicity ($\omega$)	1.2564
Softness (S)	0.1981
Nucleophilicity (N)	2.7067
Electrodonating power ($\omega ^-$)	4.6093
Electroaccepting power ($\omega ^+$)	1.0476
Net electrophilicity ($\Delta \omega ^\pm$)	5.6558

The electronegativity ($\chi$) and global hardness ($\eta$) are absolute values for the chemical reactivity that have no experimental counterpart. Indeed, they can be estimated by resorting to the experimental vertical ionization energy (I) and vertical electron affinity (A). However, these values are not known for the molecule under study. Going back to the original studies of Robert G Parr and Ralph G Pearson, some kind of classification was done in terms of the HASB principle. Anyhow, this was done only for atoms, ions or very small molecules, for which experimental values for I and A were available at this time. For molecules, of the size of the one that we are studying through this research, no standard or experimental values exist. It can only be said something about their global reactivity by comparing with other molecules of the same size. Following this criteria, when comparing with the values of the hardness of some peptides that have been studied recently^17–25^, it can be said that Aspergillipeptide D will be a bit less reactive than those used for comparison because it global hardness value is larger. A different thing can be said about the electrophilicity $\omega$ and the Nucleophilicity (N). The electrophilicity $\omega$ index encompasses the equilibrium between the tendency of an electrophile to acquire extra electron density and its resistance to exchange electron density with the environment^56^. By studying the electrophilicities of a series of reagents involved in Diels–Alder reactions^54,81,82^, and for the classification of organic compounds as strong, moderate, or marginal electrophiles, an electrophilicity $\omega$ scale was established, with $\omega$ larger than 1.5 eV for the first instance, with $\omega$ between 0.8 and 1.5 eV for the second case, and *omega* smaller than 0.8 eV for the final case^54,81,82^. By checking Table 5, it can be said that Aspergillipeptide D may be regarded as a moderate electrophile. Domingo and his collaborators^52–56^ have also proposed a Nucleophilicity index N through the consideration of the HOMO energy obtained through the KS scheme with an arbitrary shift of the origin taking the molecule of tetracyanoethylene (TCE) as a reference. An analysis of a series of common nucleophilic species participating in polar organic reactions allowed them to establish a further classification of organic molecules as strong nucleophiles with N > 3.0 eV, moderate nucleophiles with 2.0 <N < 3.0 eV and marginal nucleophiles with N < 2.0 eV. By checking again Table 5, it can be concluded that Aspergillipeptide D may be considered as a moderate nucleophile.

The global descriptors demonstrate the chemical reactivity of a each molecule in its entirety; therefore, local reactivity descriptors have been designed to assess the differences in the chemical reactivity between the areas inside a molecule. The Nucleophilic and Electrophilic Fukui functions (NFF and EFF)^31–33^ and the Dual Descriptor DD^83–88^ are some of these local reactivity descriptors. They have been defined as: NFF = $\rho _{N+1}({\mathbf {r}})-\rho _{N}({\mathbf {r}})$, EFF = $\rho _{N}({\mathbf {r}})-\rho _{N-1}({\mathbf {r}})$ and DD = $\left( {\partial \,f({\mathbf {r}})}/{\partial \,N}\right) _{\upsilon ({\mathbf {r}})}$, establishing links between the electronic densities of the various species as well as between the NFF and EFF.

**Figure 5:**
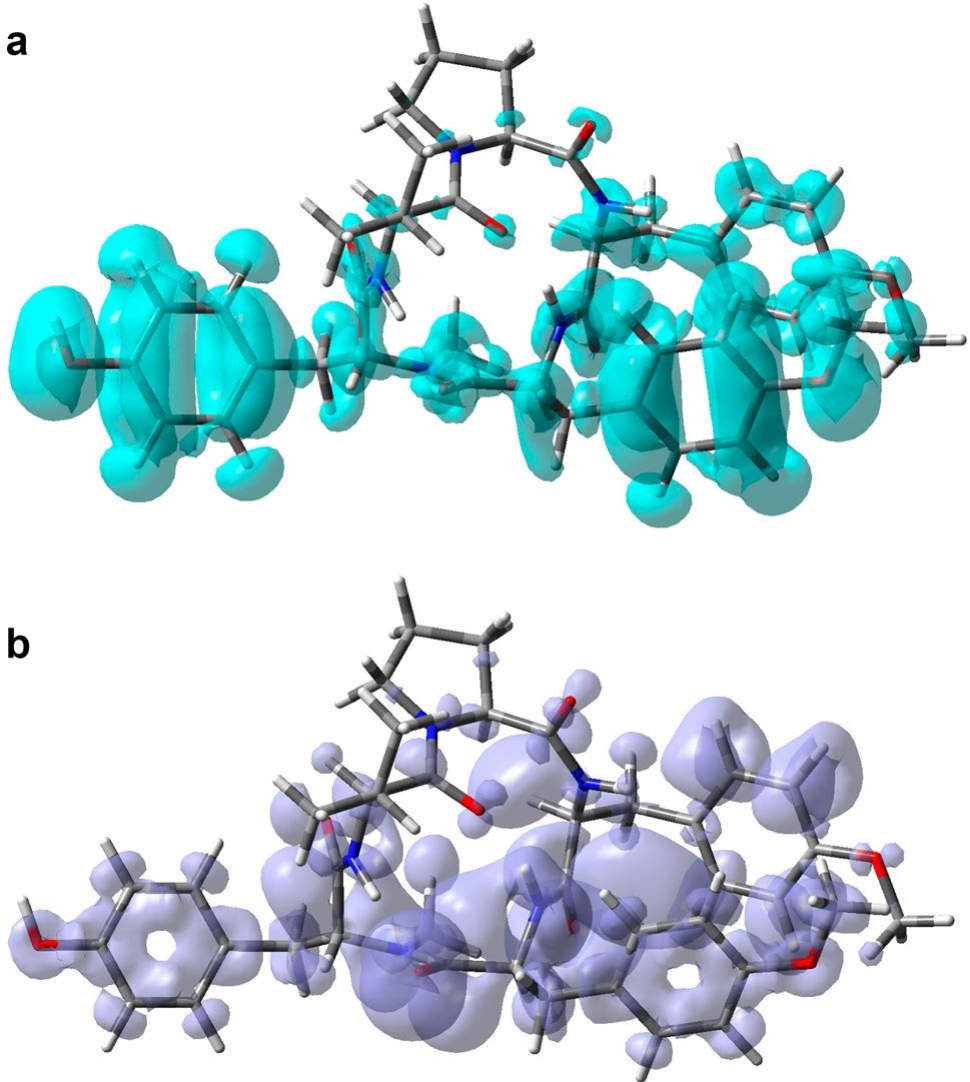
Graphical representation of the dual descriptor DD of Aspergillipeptide D. Top: DD > 0, Bottom: DD < 0.

The NFF identifies molecular locations that are more vulnerable to nucleophilic attacks, whereas the EFF identifies regions that are more vulnerable to electrophilic attacks. The reactive locations have been successfully identified using these local reactivity characteristics. However, the Dual Descriptor DD has been discovered to be capable of describing both nucleophilic and electrophilic locations within a molecule without ambiguity^88^. Figure 5 shows a graphical sketch of the Dual Descriptor DD for the Aspergillipeptide D marine cyclopentapeptide, highlighting the locations where DD > 0 and DD < 0 for a better understanding of these molecules’ local chemical reactivity.

**Table 6:** Comparison of several reactivity descriptors: Condensed Electrophilicity $\omega _k$, Condensed Nucleophilicity $N_k$ and Condensed Dual Descriptor $\Delta$f$_k$, over the atoms of the Aspergillipeptide D marine cyclopentapeptide.

Atom	$\omega _k$	$N_k$	P$_k^+$	P$_k^-$	$\Delta$f$_k$
C (1)	3.10	0.51	2.73	0.01	2.26
C (2)	1.20	1.95	0.81	0.03	0.30
N (3)	2.34	1.74	1.09	0.02	1.26
C (4)	7.47	1.41	7.41	0.01	5.40
C (5)	1.83	1.97	1.51	0.46	0.79
N (6)	2.49	0.11	2.82	0.21	1.99
C (7)	7.41	0.67	8.20	0.04	5.60
C (8)	1.96	0.37	3.20	0.02	1.42
N (9)	0.36	0.03	1.04	0.02	0.28
C (10)	0.89	0.17	1.52	0.00	0.64
C (11)	0.63	0.08	1.17	0.00	0.47
N (12)	0.47	0.16	0.18	0.00	0.31
C (13)	0.74	0.09	0.30	0.00	0.55
C (14)	0.42	0.22	0.18	0.00	0.26
N (15)	1.35	0.67	0.73	0.01	0.84
C (16)	1.16	0.45	1.56	0.00	0.76
C (17)	1.69	1.94	1.23	0.02	0.68
C (18)	4.01	1.38	9.75	0.29	2.69
C (19)	4.39	1.58	9.67	0.02	2.93
C (20)	1.76	2.34	0.99	0.19	0.61
C (21)	3.86	1.86	9.36	0.33	2.42
C (22)	4.11	1.08	8.26	0.13	2.88
O (23)	0.82	1.95	0.07	0.03	0.00
C (24)	0.38	0.84	0.44	0.21	0.03
O (25)	7.19	1.25	4.85	0.04	5.24
C (26)	0.76	2.74	0.58	5.66	− 0.30
C (27)	0.45	15.63	0.70	23.66	− 4.80
C (28)	1.01	8.03	3.33	0.67	− 1.85
C (29)	0.73	12.22	1.78	12.74	− 3.45
C (30)	0.61	14.17	0.43	18.10	− 4.19
C (31)	1.01	10.12	2.58	6.65	− 2.54
C (32)	1.01	9.22	1.10	6.57	− 2.25
O (33)	0.30	14.83	0.15	20.22	− 4.65
C (34)	0.19	3.41	0.09	2.78	− 0.97
O (35)	6.92	2.62	3.89	0.26	4.57
C (36)	1.16	0.79	0.99	0.03	0.65
C (37)	0.52	2.88	0.23	0.03	− 0.54
C (38)	0.30	16.47	0.12	0.13	− 5.19
C (39)	0.74	8.92	0.36	0.03	− 2.36
C (40)	0.78	12.23	0.45	0.05	− 3.41
C (41)	0.46	16.26	0.04	0.11	− 5.00
C (42)	0.79	12.18	0.05	0.04	− 3.40
C (43)	0.82	9.31	0.38	0.02	− 2.43
O (44)	0.28	18.45	0.00	0.09	− 5.86
O (45)	3.39	1.62	1.19	0.02	2.13
C (46)	0.14	0.07	0.05	0.00	0.08
C (47)	0.14	0.09	0.05	0.00	0.08
C (48)	0.15	0.06	0.05	0.00	0.10
O (49)	0.88	0.11	0.53	0.00	0.66
C (50)	0.30	0.07	0.43	0.00	0.22
C (51)	0.19	0.06	0.17	0.00	0.13
C (52)	0.25	0.08	0.12	0.00	0.17
O (53)	1.39	0.38	0.55	0.00	0.97

H atoms are not shown.

**Figure 6:**
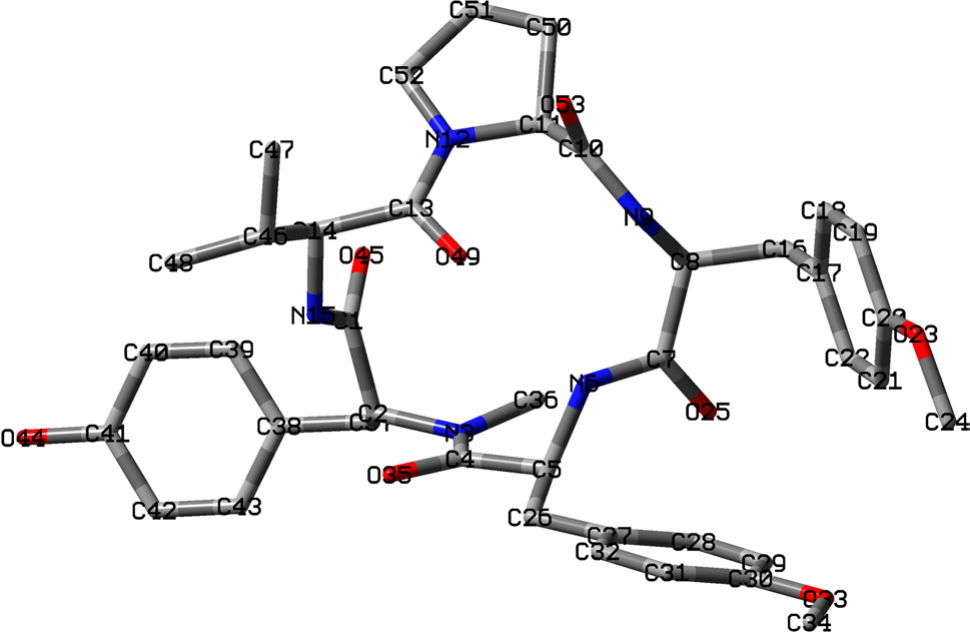
Schematic representation of Aspergillipeptide D showing the labels for the atom types and numbering.

As a result, we have decided to compare the results of the Condensed Dual Descriptor $\Delta$f$_k$ calculated from either Hirshfeld Population Analysis (HPA) with the Condensed Electrophilicity $\omega _k$ and Condensed Nucleophilicity $N_k$ descriptors^89,90^ calculated from the same HPA over all atoms in the molecule, with the exception of the Hs. Table 6 presents a comparison of several reactivity descriptors: Condensed Electrophilicity $\omega _k$, Condensed Nucleophilicity $N_k$ and Condensed Dual Descriptor $\Delta$f$_k$ over selected atoms of the Aspergillipeptide D marine cyclopentapeptide in relation with Fig. 6 that displays an schematic representation of the molecule showing the labels for the selected atoms:

## Conclusions

By considering our suggested computational peptidology methodology, the Aspergillipeptide D marine cyclopentapeptide isolated from marine sources has been studied by applying certain methods generally used in the procedure of drug discovery and development, showing that this peptide may be regarded as a potential therapeutic drug.

The biological targets, physicochemical attributes, and ADMET (Absorption, Distribution, Metabolism, Excretion, and Toxicity) indices associated with the bioavailability and pharmacokinetics of the marine cyclopentapeptide being studied were forecasted and analyzed as descriptors that could be useful in future QSAR studies.

With this knowledge, the chemical reactivity of the Aspergillipeptide D marine cyclopentapeptide has been thoroughly investigated by optimizing their structures using DFTBA methodology and calculating their electronic properties using a high-quality model chemistry, namely MN12SX/Def2TZVP/H2O, which has already been used in previous research, demonstrating its utility for this type of calculation. As for the case of previous studies on the chemical reactivity of marine cyclopeptides, this work represents a confirmation of the superiority of the MN12SX density functionals over other long-range corrected density functionals because it allowed the estimation of the frontier orbital energies with great accuracy based on the KID procedure evaluation. The fact that the energy of the LUMO and of the SOMO (or the HOMO energy of the anion) are almost the same, which is reflected in the KID accuracy descriptor $\Delta$SL being very close to zero, is an indication that the derivative discontinuity is negligible for the chosen density functional. This is translated as the ability of the LUMO energy to reflect with precision the Electron Affinity of the molecule, implying that the chemical reactivity parameters obtained by considering this density functional will be very accurate. This is a very important result because it allowed the estimation of the accuracy of the calculation only based on the fulfilment of some intrinsic requirements (like the Janak and Ionization Energies) without the need to resort to the comparison with experimental results that could not be available, as in the present case.
